# Phylogenetic analyses, protein modeling and active site prediction of two pathogenesis related (PR2 and PR3) genes from bread wheat

**DOI:** 10.1371/journal.pone.0257392

**Published:** 2021-09-10

**Authors:** Muhammad Numan, Shazia Anwer Bukhari, Mahmood-ur- Rehman, Ghulam Mustafa, Bushra Sadia

**Affiliations:** 1 Department of Biochemistry, Government College University, Faisalabad, Pakistan; 2 Department of Bioinformatics and Biotechnology, Government College University, Faisalabad, Pakistan; 3 Centre of Agricultural Biochemistry and Biotechnology (CABB), University of Agriculture, Faisalabad, Pakistan; University of Nebraska-Lincoln, UNITED STATES

## Abstract

Wheat is a major staple food and has been extensively grown around the globe. Sessile nature of plants has exposed them to a lot of biotic and abiotic stresses including fungal pathogen attack. *Puccinia graminis* f.sp. *tritici* causes stem rust in the wheat crop and leads to 70% decrease in its production. Pathogenesis-related (PR) proteins provide plants with defense against different fungal pathogens as these proteins have antifungal activities. This study was designed to screen Pakistani wheat varieties for PR2 and PR3 proteins and their *in silico* characterization. PR2 and PR3 genes were screened and isolated by PCR amplification from wheat variety Chenab-70 and Frontana, respectively. The nucleotide sequences of PR2 and PR3 genes were deposited in GenBank with accession numbers MT303867 and MZ766118, respectively. Physicochemical properties, secondary and tertiary structure predictions, and molecular docking of protein sequences of PR2 and PR3 were performed using different bioinformatics tools and software. PR2 and PR3 genes were identified to encode β–1,3–glucanase and chitinase proteins, respectively. Molecular docking of both PR2 and PR3 proteins with beta-glucan and chitin (i.e. their respective ligands) showed crucial amino acid residues involved in molecular interactions. Conclusively, molecular docking analysis of β–1,3–glucanase and chitinase proteins revealed crucial amino acid residues which are involved in ligand binding and important interactions which might have important role in plant defense against fungal pathogens. Moreover, the active residues in the active sties of these proteins can be identified through mutational studies and resulting information might help understanding how these proteins are involved in plant defense mechanisms.

## Introduction

Wheat (*Triticum aestivum* L.) is the most important and a major staple food being grown in approximately 89 countries of the world. The world population, including humans and livestock, has been increasing continuously which has increased the food demand proportionally that has ultimately increased the production of high-yielding and stress-tolerant wheat varieties [1, 2]. According to the World Urbanization Prospect of United Nations, the current population of Pakistan is 220.9 M with the increased growth rate of 2.0% in 2020 [3]. The increased population has developed a huge gap between lower wheat production and higher consumer demand for food.

Wheat being sessile in nature is continuously exposed to various fungal pathogens [4]. *Puccinia graminis* f.sp. *tritici* is a major fungal pathogen which causes stem rust and decreases the wheat production up to 70% approximately [5]. Plants employ several genes to provide defense against fungal attacks by activating innate and systemic acquired resistance mechanisms [6]. Among those several defense-related genes, pathogenesis-related (PR) gene family provides plants with the best defense against fungal pathogens. PR gene family contains 17 different classes of PR genes [7, 8]. Among 17 gene classes, PR2 and PR3 genes are reported to encode β–1,3–glucanase and chitinase, respectively [7, 9, 10].

β–1,3–glucans are the polymers of glucose and make up to 60–90% of fungal cell wall. β–1,3–glucanase breaks down the fungal cell wall by degrading β–1,3 glycosidic linkage presented between glucose residues of β–1,3–glucans and provides defense to the plants [9, 11]. Chitin is also a polymer of glucose residues with β–1,4 glycosidic linkage and abundant in plant cell wall [12]. Chitinase enzyme hydrolyzes the β–1,4 glycosidic linkage present in chitin polymer and provides defense to plants against fungal pathogens [8, 13]. β–1,3–glucanase and chitinase proteins are reported as best soldiers against fungal pathogens. In addition to plants, β–1,3–glucanase and chitinase proteins produced by bacteria [14] and fungi [15] also show excellent antifungal activities.

*In silico* studies of both β–1,3–glucanase and chitinase proteins from bacteria, fungi and different plants have been reported [12, 15–17]. However, there is a lack of *in silico* characterization and molecular interactions of both β–1,3–glucanase and chitinase proteins from wheat and their respective ligands in literature. The aim of this study was therefore to screen and identify PR2 and PR3 genes from Pakistani wheat varieties and their respective sequences. Furthermore, *in silico* characterization and molecular docking of both β–1,3–glucanase and chitinase proteins with their ligands (i.e., beta glucan and chitin) were performed through different bioinformatics tools and software. We found important amino acid residues which are involved in the molecular interactions in active sites of both β–1,3–glucanase and chitinase proteins.

## Materials and methods

### Seed collection and plant growth

Seeds of nine different wheat varieties were collected from the Ayub Agricultural Research Institute, Faisalabad, Pakistan and sown in 4-inch pots in the Molecular and Medical Genetics Laboratory (MMGL), the Department of Biochemistry, Government College University, Faisalabad, Pakistan.

### RNA extraction and screening of pathogenesis-related gene(s)

The total RNA was extracted using GeneJet RNA Purification Kit (ThermoScientific, USA; Catalog # K0731) from the fresh leaves of wheat plants [18]. Complimentary DNA (cDNA) template was synthesized by using RevertAid First strand cDNA synthesis kit (ThermoScientific, USA; Catalog # K1621) from all purified RNA samples. The PR2 and PR3 genes were amplified from cDNA template through polymerase chain reaction (PCR) and the amplicons were gel purified.

### Phylogenetic tree construction

The purified PCR products were sequenced using commercial services of the Eurofins Genomics, USA. The contigs were made using DNA Dragon software version 1.6.0 (SequentiX–Digital DNA Processing, Germany). All the assembled contigs (nucleotide sequences) of PR2 and PR3 genes were translated into protein using online bioinformatics tool (i.e. ExPASy–Translate (https://web.expasy.org/translate/)). Amino acid sequences of PR2 and PR3 proteins were used to search homologs by basic local alignment search tool for proteins (BLASTp) [19] from reference sequence (Refseq) database [20]. Phylogenetic trees for PR2 and PR3 proteins were reconstructed with MEGA-X software (Version 10.2.0) using Neighbor-Joining method with 1000 bootstrap value and p-distance as substitution model as described previously with slight modifications [21].

### *In silico* characterization

The physicochemical parameters such as amino acid composition, theoretical isoelectric point (pI), molecular weight, extinction coefficient, instability index, aliphatic index and total number of positively and negatively charged amino acids were obtained using ProtParam [22]. Secondary structures were predicted using SOPMA [23], GOR4 [24] and HNN [25] servers while domain analysis was performed using pfam database [26].

### 3D model prediction and evaluation

The homology modeling approach was used to predict 3D structures of PR proteins and SWISS-Model online server was used for this purpose [27]. Ramachandran plots were built through PROCHECK [28] to analyze correct stereochemistry of the predicted models. Furthermore, all the predicted models were structurally and energetically verified by different model evaluation tools such as protein structure analysis (ProSA) server, ERRAT [29] and Verify3D [30].

### Evaluation of ligand interactions with receptor proteins

The interactions of ligands with their respective receptor proteins were explored by molecular docking approach using Molecular Operating Environment (MOE) software (v2014.0.1.9) [31]. The ligand structures i.e., β-glucan (PubChem ID: 71312131) and chitin (PubChem ID: 6857375) were retrieved in.sdf format from PubChem database [32, 33] and saved in the MOE database after energy minimization. The docking algorithm of MOE was used to dock prepared ligand database with the active site of the receptor protein. The siteFINDER tool of MOE was used to find the binding residues with default parameters such as rescoring 1: London dG, retain: 10, refinement: force field, rescoring 2: London dG, and retain: 10.

## Results

### Seed collection and plant growth

Seeds of nine different wheat varieties were sown in 4ʺ plastic pots and kept in the plant growth chamber up to three leaf stage. All the wheat varieties used in this study are shown in S1 Fig in S1 File.

### RNA extraction and screening of PR proteins

**Fig 1:**
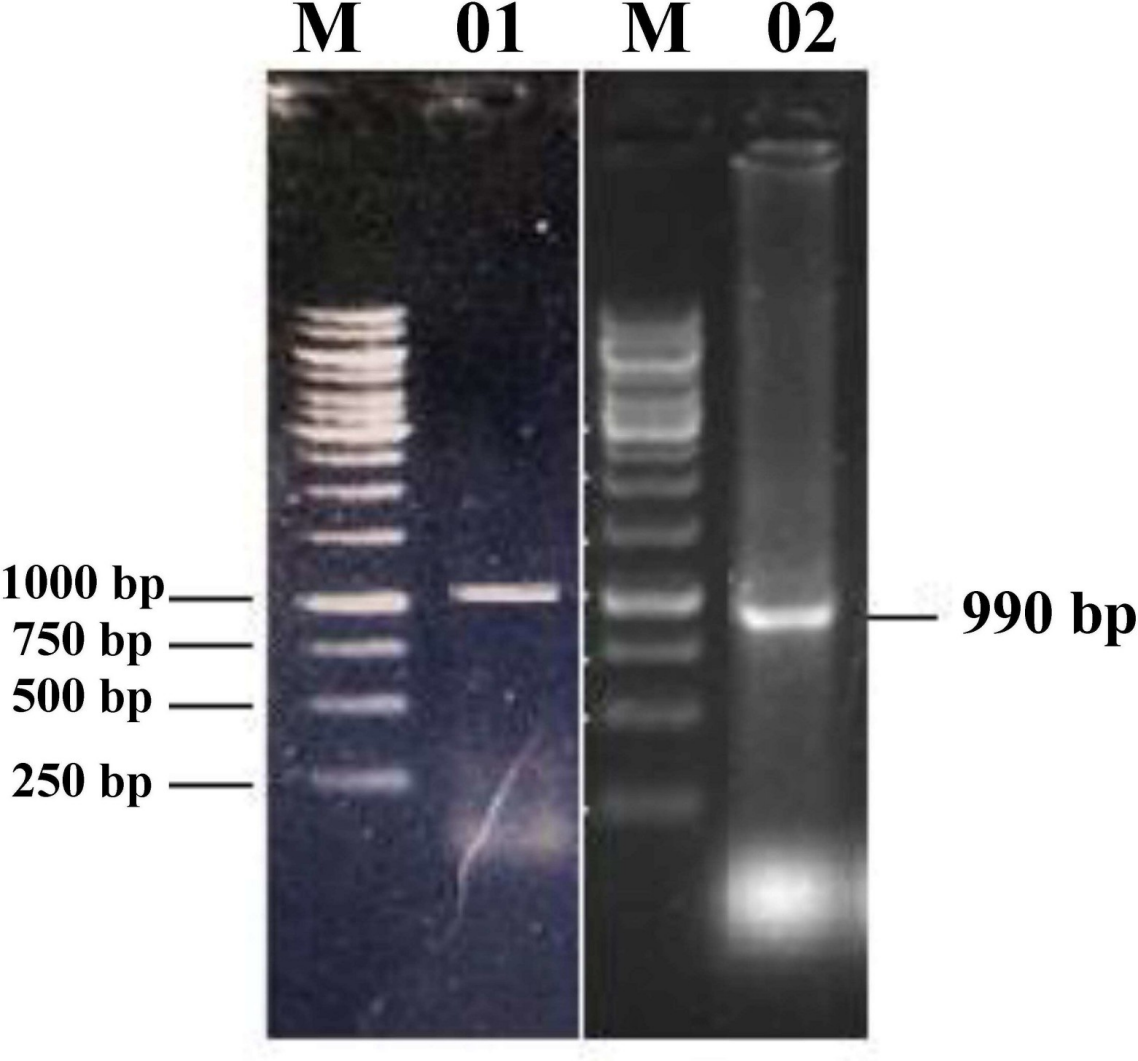
Full-length gene amplifications of PR2 and PR3 genes. M = 1 kb DNA ladder, 01 = PR2 gene from wheat variety Chenab-70 and 02 = PR3 gene from wheat variety Frontana.

RNA from the selected wheat varieties was extracted (S2 Fig in S1 File) and PCR was done to amplify PR2 and PR3 genes from cDNA template using previously designed primers (S1 Table in S1 File). The Fig 1 shows the clear bands and approximate lengths of PR2 and PR3 genes. PR2 gene was amplified only in the wheat variety Chenab-70 (S3a Fig in S1 File) and PR3 in the wheat variety Frontana (S3b Fig in S1 File).

### Phylogenetic tree construction

**Fig 2:**
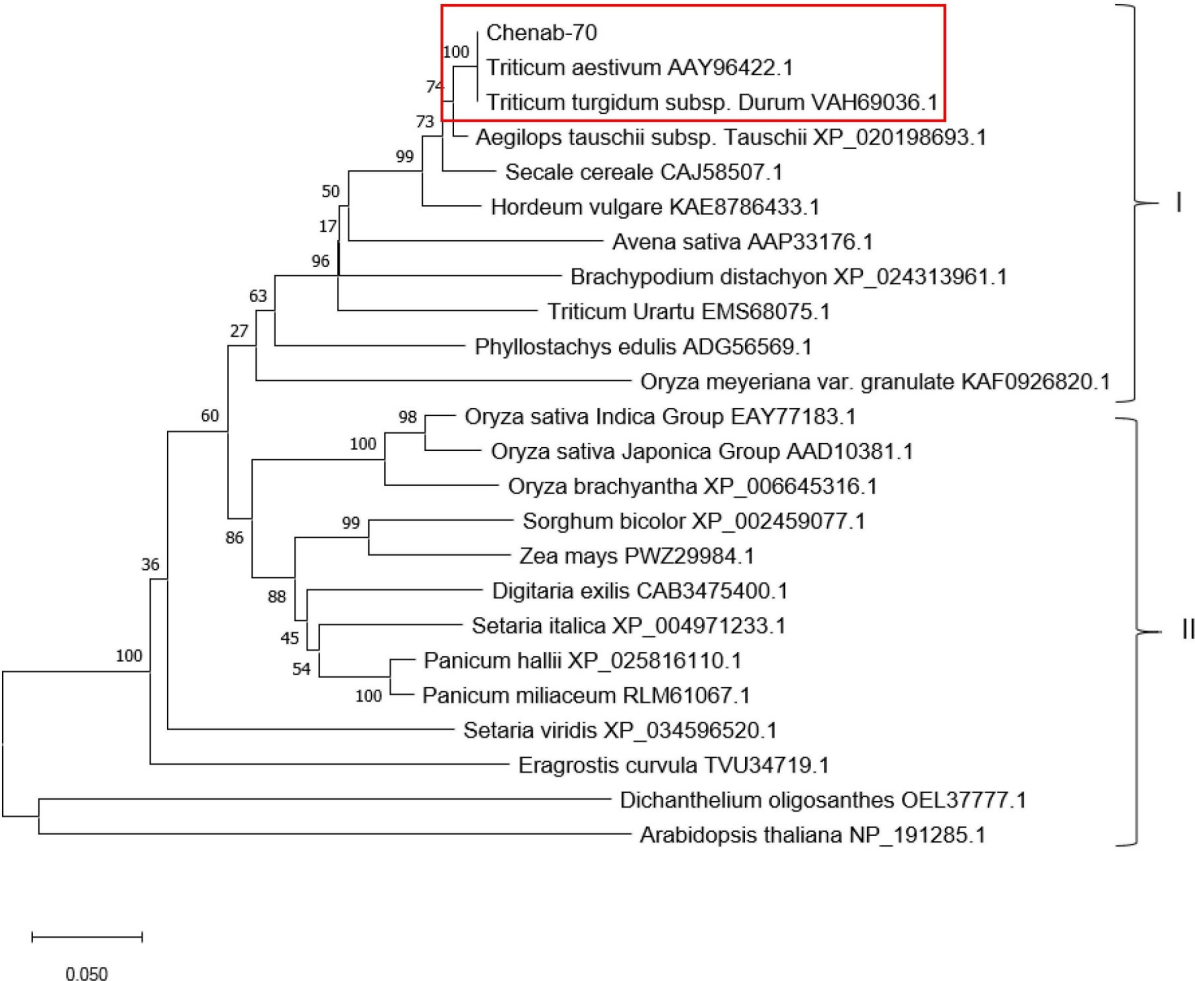
Phylogenetic tree of PR2 protein and its selected homologs. Phylogenetic tree was reconstructed with bootstrap method using 1000 bootstrap replications and p-distance was employed as substitutional model with substitution type as amino acids and rates and patterns were kept uniform. For phylogenetic tree reconstruction, the PR2 protein sequences of different members of the Poaceae family were used. Our protein sequence was clustered with the *Triticum aestivum* spp. and showed a close relationship with AAY96422.1 than any other member of the family in the same lineage.

**Fig 3:**
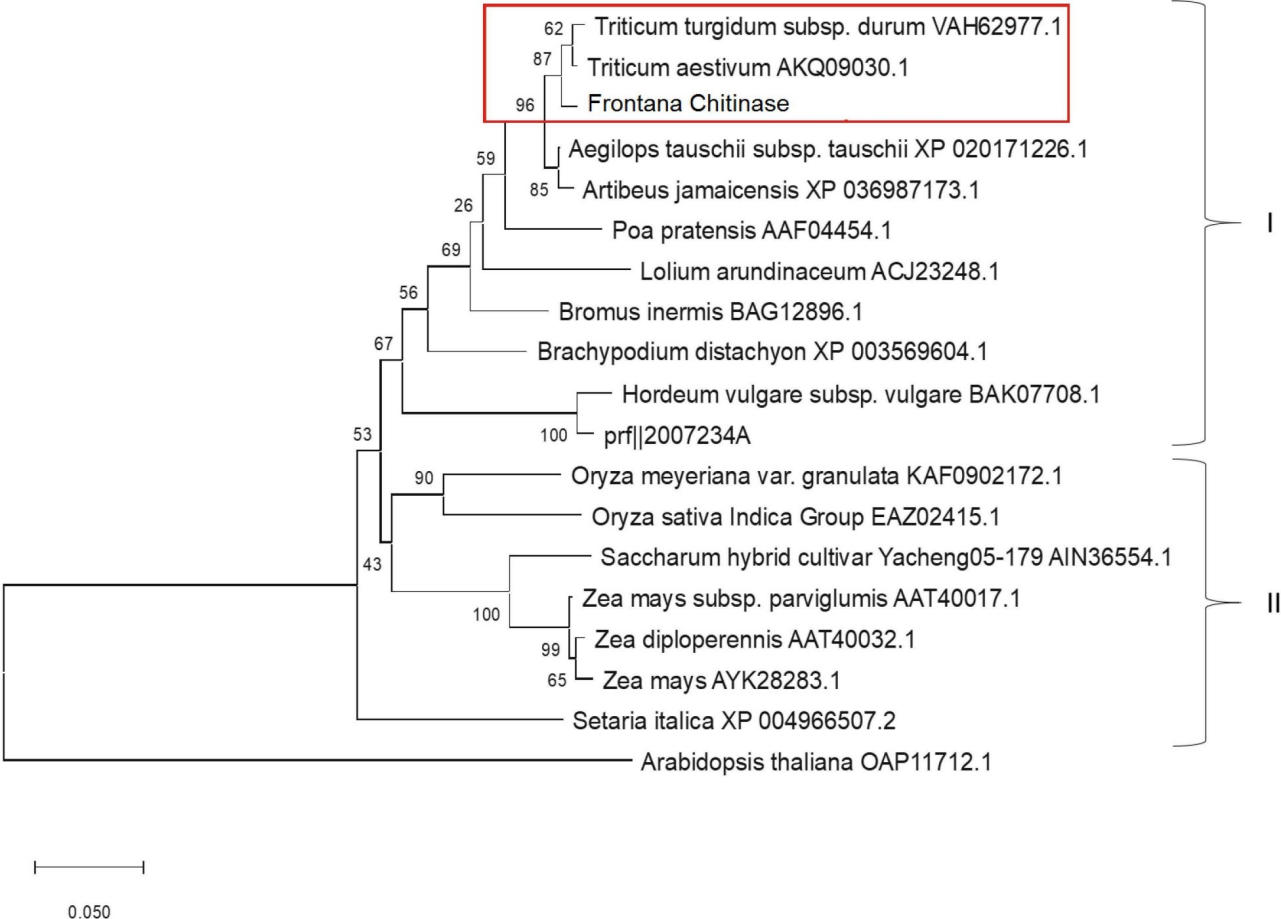
Phylogenetic tree of PR3 protein and its selected homologs. Phylogenetic tree was reconstructed with bootstrap method using 1000 bootstrap replications and p-distance was employed as substitutional model with substitution type as amino acids and rates and patterns were kept uniform. For phylogenetic tree reconstruction, the PR3 protein sequences of different members of the Poaceae family were used. Our protein sequence was clustered with the *Triticum aestivum* spp. and showed a close relationship with AKQ09030.1 than any other member of the family in the same lineage.

The sequenced forward and reverse fragments of PR2 and PR3 genes were assembled into two separate contigs to obtain full-length gene sequences. The sequences of PR2 and PR3 genes were found to be 1076 and 1012 nucleotides long, respectively. After careful analyses, the mRNA sequences of PR2 and PR3 genes were submitted to GenBank and allotted with accession numbers: MT303867 and MZ766118, respectively. Phylogenetic trees of both genes were reconstructed using their respective protein sequences. The phylogenetic trees of both proteins were divided into two clusters i.e., cluster I and cluster II. Protein sequence of PR2 was clustered with *T*. *aestivum* spp. and more closely related to the accession number AAY96422 (Fig 2) and the protein sequence of PR3 was also clustered with *T*. *aestivum* spp. and showed a close evolutionary relationship with AKQ09030 that any other member of the family as shown in red rectangle in Fig 3.

### Physicochemical properties, secondary structure and protein domain prediction

Physicochemical properties of both proteins were predicted from online server ProtParam. Both proteins were predicted to be small with molecular weights of 35.35 kDa and 33.5 kDa, respectively. PR2 protein was predicted to be basic in nature with pI of 8.50 while PR3 was slightly acidic in nature with pI of 6.89 (Table 1).

**Table 1:** Physicochemical properties of PR2 and PR3 proteins predicted by ProtParam.

Physicochemical parameters	PR2	PR3
Number of AA	334	319
Molecular weight (d)	35355.86	33525.36
Theoretical pI	8.50	6.89
Negatively charged AA (n)	22	21
Positively charged AA (n)	24	21
Extinction coefficients (M^-1^cm^-1^)	34840	53860
Instability index (II)	33.76	37.76
Aliphatic Index	84.52	57.27
Grand average of hydropathicity (GRAVY)	0.028	-0.200

The secondary structures of both proteins were predicted by SOPMA, GOR4 and HNN servers which showed that random coils were predominantly present in PR2 (39.22–50.60%) and PR3 (56.74–65.20%) proteins (Table 2).

**Table 2:** Secondary structure analyses of PR2 and PR3 proteins by different servers.

Gene	Server	Alpha helices		Extended strands		Random coils		Beta turns	
		No. of residues	%age	No. of residues	%age	No. of residues	%age	No. of residues	%age
PR 2	HNN	129	38.62	50	14.97	155	46.41	-	-
	SOPMA	117	35.03	65	19.46	131	39.22	21	6.29
	GOR4	106	31.74	59	17.66	169	50.6	-	-
PR 3	HNN	85	26.65	26	8.15	208	65.2	-	-
	SOPMA	75	23.51	42	13.17	181	56.74	21	6.58
	GOR4	50	15.67	63	19.75	206	64.58	-	-

Protein domain analysis was performed using Hidden Markov Model (HMM) through Pfam. The PR2 protein sequence showed a significant match to HMM with E-value of 8.8e^-105^ and found to be a member of the glycosyl hydrolases family (Glyco Hydro 17) (S4A Fig in S1 File). The protein sequence of PR3 showed a total of four matches (i.e., two of which were significant while other two were found to be non-significant). The HMM match with the lowest E-value of 3.0e^-130^ was selected and found to be the member of the chitinase class I family (Glyco hydro 19) (S4B Fig in S1 File).

### Homology modeling

**Fig 4:**
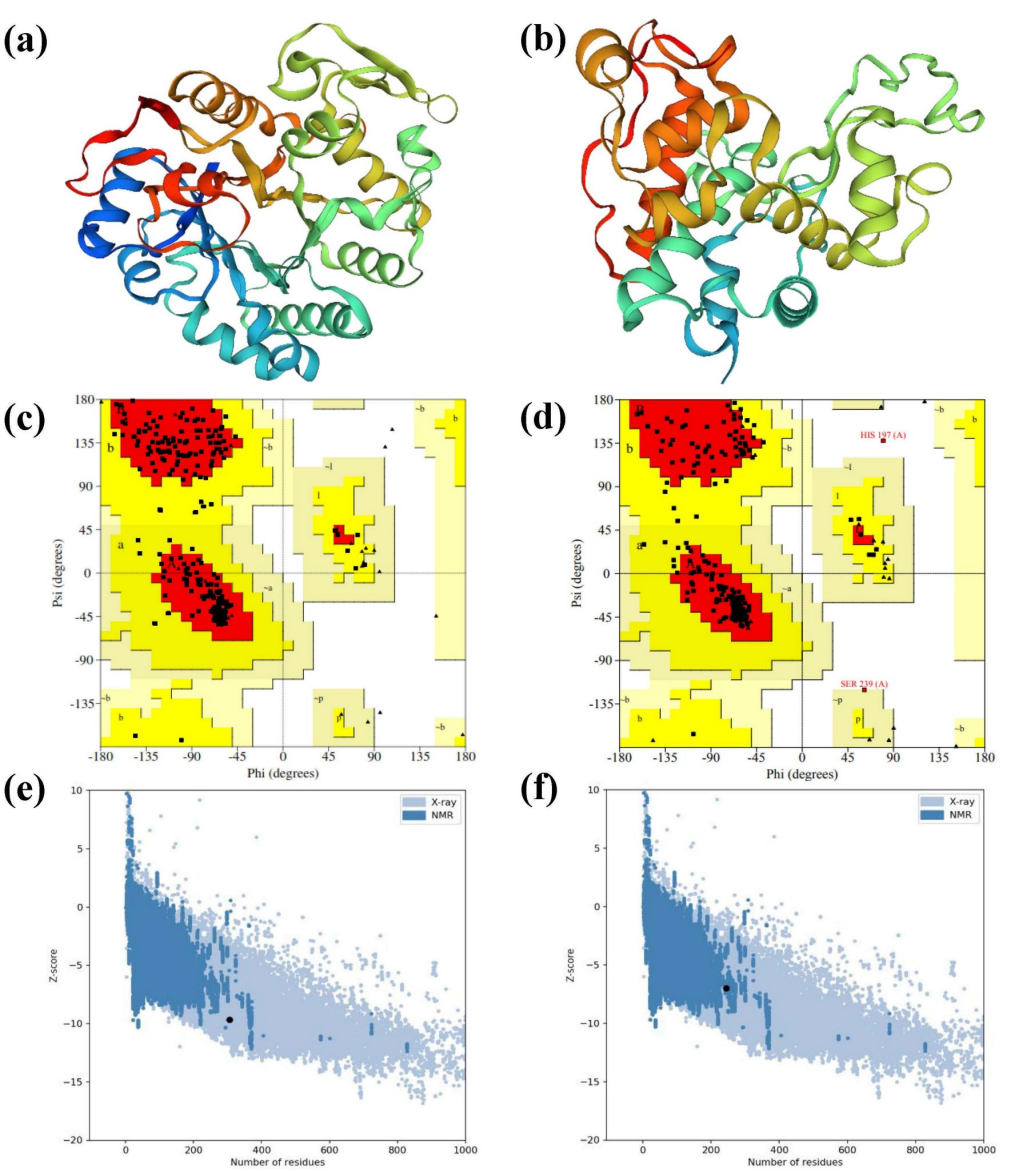
Predicted 3D models of PR2 and PR3 proteins and their evaluations. 3D structures of PR2 and PR3 proteins, respectively (a, b); Ramachandran plots of PR2 and PR3 showing amino acid placement in allowed and disallowed regions of plots (c, d); Z-score of both PR2 and PR3 proteins is shown which is the indicator of overall quality of predicted 3D model (e, f).

To study the structural arrangement of both proteins, 3D models were built by an online server SWISS-Model. SWISS-Model used BLAST [34] and HHblits [35] to align the target sequence with previously characterized sequences and searches for the best template(s). The best selected templates were 1ghs.1.A and 1cns.1.A for PR2 and PR3 proteins with maximum sequence identities (95.10% and 81.40%), coverage (92% and 76%) for PR2 and PR3, respectively (Fig 4A and 4B). The range of the predicted model of PR2 protein was from amino acid 29 to 334 and for PR3 the range was from amino acid 76 to 319. The values of root-mean-square deviation (RMSD) for PR2 and PR3 with their respective templates were found to be 0.068 and 0.081, respectively. The stereochemistry of both models was validated by building the Ramachandran plots for amino acids in core, additionally allowed, generously allowed and disallowed regions (Table 3).

**Table 3:** PROCHECK Ramachandran statistics of predicted 3D models of PR2 and PR3 proteins.

Variable	PR2		PR3	
	No. of residues	Percentage	No. of residues	Percentage
Most favored region	237	90.8	171	86.4
Additionally allowed region	24	9.2	25	12.6
Generously allowed region	0	0	1	0.5
Disallowed	0	0	1	0.5
Non-glycine and non-proline residues	261	100	198	100
Number of end-residues (exc. Gly and Pro)	2		1	
Number of glycine residues (triangles)	28		30	
Number of proline residues	15		15	
Total residues	306		244	

Ramachandran plot of PR2 protein had 90.8% of amino acids in the core region and no amino acid was appeared in the disallowed region while PR3 protein had 86.4% amino acids in the core region, one in generously allowed and one in disallowed region (Fig 4C and 4D). The overall protein structure quality was measured by z-plot for both predicted models using ProSA. The overall quality of both models is represented by the Z-scores of -9.67 and -6.99, respectively. The Z-score of predicted 3D model of PR2 was located within the space of X-ray, while the Z-score of 3D model of PR3 protein was present within the space of NMR protein structure (Fig 4E and 4F).

**Fig 5:**
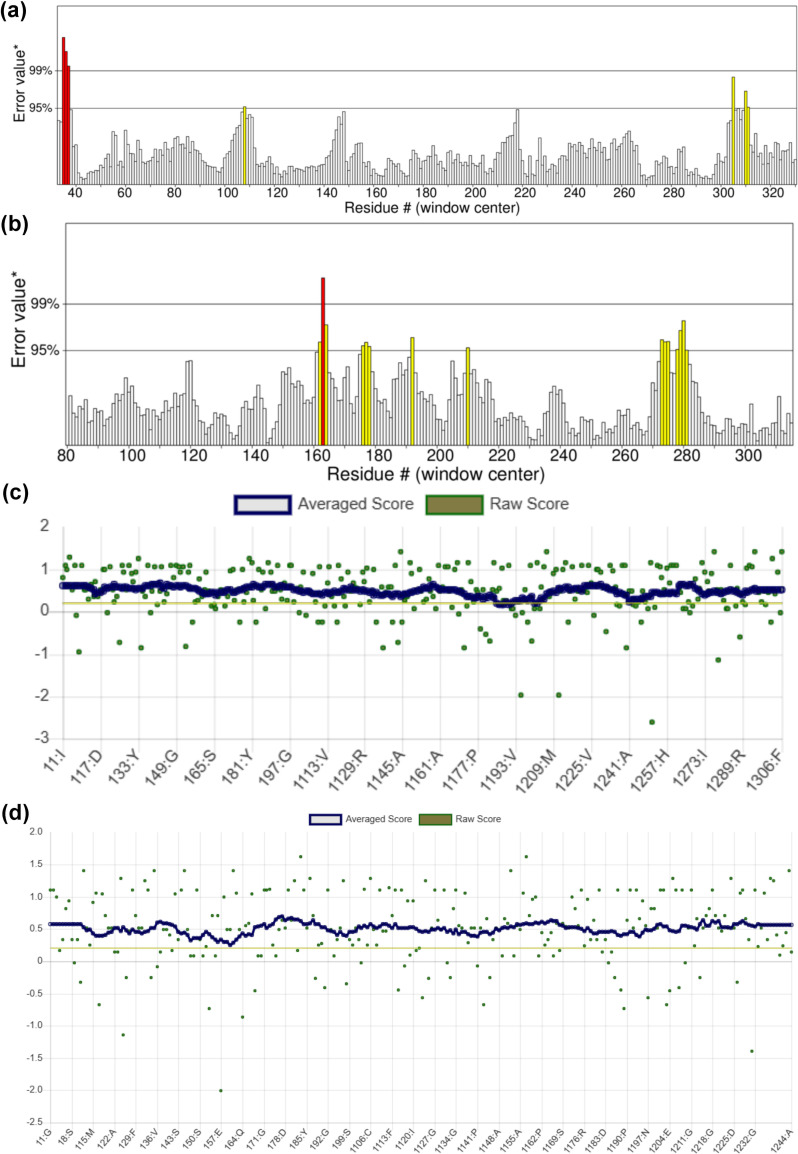
Evaluation of predicted 3D models of PR2 and PR3 proteins by ERRAT and Verify3D online servers. ERRAT graphs of both PR2 and PR3 protein models are showing the correctly and incorrectly determined regions of proteins structures. Two lines (error bars) showed the confidence with which incorrectly determined regions can be rejected. A good and high-resolution structure produces value around 95% or more (A, B). More than 80% of amino acids have shown overall structure correctness value above 0.2 which is an indicator of the correctness of 3D structures of both PR2 and PR3 protein models (C, D).

To investigate the correctly and incorrectly determined regions and overall correctness of the 3D models, the models were validated through online servers ERRAT and Verify3D. ERRAT calculated the overall quality factor of 97.65 and 93.62 for PR2 and PR3, respectively (Fig 5A and 5B). Verify3D calculated 98.04% and 100% of the amino acid residues for averaged 3D-1D score of ≥0.2 of PR2 and PR3 3D models, respectively (Fig 5C and 5D). From the Verify3D results, as the cut-off scores were ≥0.2 therefore, these imply the predicted models are valid. The results of both servers duly verified the predicted models of PR2 and PR3 proteins.

On the axis of error value, the two lines are indicating the confidence with which it is possible to reject the regions that exceed that error value. The overall quality factor is determined as the percentage of the protein for which the calculated error value falls below the 95% rejection limit. Good high resolution structures generally produce values around 95% or higher. For lower resolution (i.e., 2.5 to 3Å) the average overall quality factor is around 91%. More than 80% of amino acids have shown overall structure correctness value >0.2, which is an indicator of the correctness of 3D structures of both PR2 and PR3 proteins (c, d).

### Molecular docking studies

**Fig 6:**
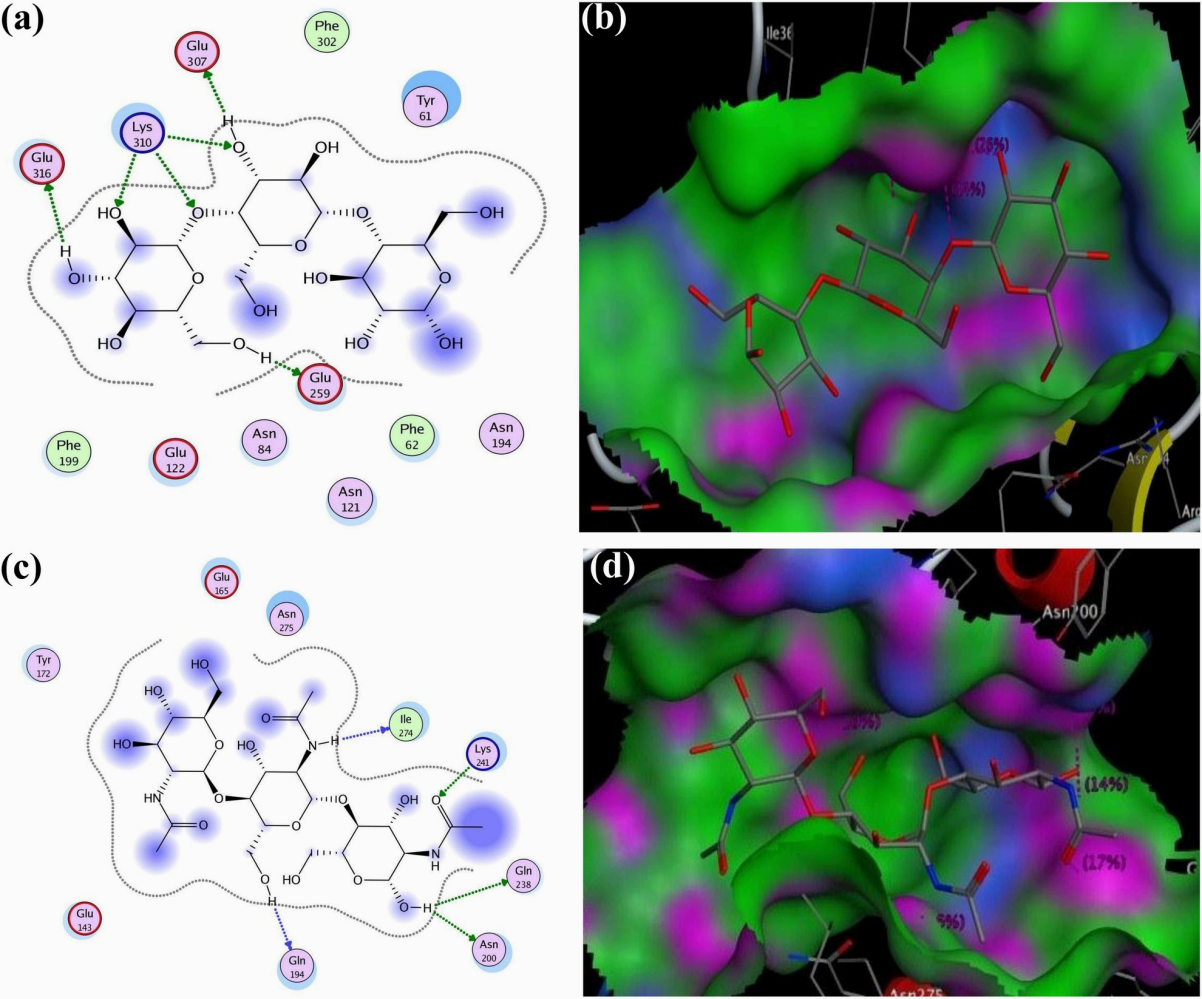
Molecular docking and interaction study of PR2 and PR3 proteins with their respective ligands. PR2 protein residues (Glu307, Glu259, Lys310 and Glu316) are showing interactions with beta-glucan. Lys310 residue was the most crucial and involved in most interactions with ligand (a); surface map of PR2 protein and ligand interaction (b). PR3 protein residues (Gln194, Asn200, Gln238, Lys241 and Ile274) are showing interactions with chitin (c); surface map of PR3 protein and ligand interactions (d). Green surface is representing the binding pocket of the receptor proteins while ligands are rendered as sticks.

To investigate the molecular interactions of β-d-glucan with PR2 protein and chitin with PR3 protein, the molecular docking was performed. The docking results of β-d-glucan with PR2 protein showed Lys310 as a crucial amino acid in the molecular interaction because it was acting as sidechain hydrogen bond donor (HBD). It interacted with three oxygen atoms (i.e., two oxygen atoms of hydroxyl groups attached to the two different rings of β-d-glucan and one oxygen joining the same two rings of β-d-glucan with ether linkage) and three glutamic acid residues (i.e., Glu259, Glu307 and Glu316) interacted as sidechain hydrogen bond acceptors (HBA) (Fig 6A). Furthermore, Tyr61, Phe62, Asn84, Asn121, Glu122, Asn194, Phe199 and Phe302 were present in the environmental space (Fig 6A). The strong binding mode of the β-d-glucan with PR2 protein is given in Fig 6B. Similarly, the interactions of chitin with PR3 as receptor protein are shown in Fig 6C and the binding mode of chitin with PR3 as target molecule is given in Fig 6D. The molecular docking results of chitin with PR3 protein showed two amino acids (i.e., Gln194 and Ile274) interacting as backbone HBAs and Lys241 acted as sidechain HBD. The Asn200 and Gln238 were acting as sidechain HBAs in the molecular interactions (Fig 6C). Furthermore, Glu143, Glu165, Tyr172, Ser196 and Asn275 were present in the environmental space (Fig 6C).

## Discussion

Crop protection and increased production is a major challenge in agricultural sciences worldwide as plants are continuously exposed to fungal pathogens. In developed countries, it is estimated that 25% of wheat crop is lost due to plant diseases while in developing countries more than 50% of the crop is lost. So, it is necessary to develop new tolerant varieties for more crop production [10, 36]. This study was focused to screen local Pakistani wheat varieties for pathogen-resistant genes and their *In-silico* analyses so that more and more information can be extracted for the development of tolerant and high-yielding wheat varieties.

In current study, two pathogenesis-related genes i.e., PR2 and PR3 were screened and identified from two Pakistani wheat varieties Chenab 70 and Frontana, respectively. Phylogenetic analyses of amino acid sequences have shown that PR2 protein from *Triticum aestivum* Chenab-70 has a strong evolutionary relationship with *Triticum aestivum* (Chitinase: AAY96422.1) (i.e., with a bootstrap value of 100) and PR3 protein from *Tritucum aestivum* Frontana was closely clustered with *Triticum aestivum* (Chitinase: AKQ09030.1) (i.e., with a bootstrap value of 87). Phylogenetic analysis has been extensively used for investigating the evolutionary relationships among related groups of taxa [10, 37].

A range of β–1,3–glucanase and chitinase proteins have been identified and their *in silico* characterization including physicochemical properties, and predictions of their secondary and tertiary structures have been done from different organisms ranging from bacteria, algae [38], fungi [39] and insects [40, 41] to plants [11, 17]. The molecular weight of β–1,3–glucanases ranges from 33–36 kDa and can be classified into three different classes (i.e., classes I, II and III). The molecular weight of class I β–1,3–glucanases is approximately 33 kDa and reported to be basic in nature while class II and III β–1,3–glucanases molecular weight ranges from 34–36 kDa and have been found to be acidic in nature [7, 42, 43]. Sinha et al. [7] has reported that chitinases also have different basic and acidic proteins with molecular weight ranges from 25–35 kDa. Furthermore, recent studies have revealed the molecular weight of chitinases ranged from 34.5 to 49.5 KDa with theoretical pI 4.81–7.94 showing acidic to slightly basic nature of chitinase genes [15, 44]. In our study, the molecular weight of β–1,3–glucanase and chitinase proteins were found to be 35.4 kDa and 33.5 kDa, respectively while theoretical pI for β–1,3–glucanase was slightly basic (i.e., 8.50) and pI for chitinase protein was slightly acidic (i.e., 6.89). On the basis of above discussion our findings are in line with previously reported results.

Present study described high proportions of random coils and α-helices in both β–1,3–glucanase and chitinase proteins. In previous studies secondary structure prediction has shown higher proportions of random coils (46.8%) and α-helices (30.4%) in *Beauveria bassiana* chitinase and β–1,3–glucanase proteins. The higher proportions of random coils and α-helices provide stability to the enzyme [15, 45, 46]. β–1,3–glucanase and chitinase proteins belong to hydrolase family which hydrolyzes the β–1,3 and β–1,4 glycosidic linkages present in β-glucans and chitin, respectively [11, 12]. In current study, the domain analysis identified β–1,3–glucanase as a member of glycosyl hydrolase family 17 and chitinase as a member of glycosyl hydrolase family 19 (S4 Fig in S1 File). The domain of β-amylase-like protein has been found to be a member of glyco hydro family 14 [47].

The 3D structure prediction contributes towards understanding of the functions of different protiens. Determination of actual crystal structure of any protein is very difficult as the process involves highly complex techniques such as nuclear magnatic resonanse and crystallography [48, 49]. Bioinformatics tools such as SWISS-Model, ProSA, PROCHECK, ERRAT, Verify3D are the best alternatives to predict and evaluate the protein 3D structures [50, 51]. In this study, 3D structures of PR2 and PR3 proteins were predicted using SWISS-Model. The Z-score serves as an indicator of overall quality of protein predicted 3D structures and determine the average energy deviation with regards to engergy distribution derived from random conformations. The Z-score is calculated by comparing the related proteins structures exerimentally resolved by X-rays or NMP present in the current protein databank [52, 53]. Furthermore, PROCHECK showes the amino acid distribution in allowed, additionally allowed and disallowed regions by means of Ramachanran plot. ERRAT and Verify3D estimate the values for overall quality factor and analyse correctly and incorrectly determined regions in the predicted 3D structures. The two lines (error bars) are showing the confidence with which incorrectly determined regions can be rejected. A good and high-resolution structure produces value around 95% or more (Fig 5A and 5B). On the axis of error value, the two lines are indicating the confidence with which it is possible to reject the regions that exceed that error value. The overall quality factor is determined as the percentage of the protein for which the calculated error value falls below the 95% rejection limit. Good high-resolution structures generally produce values around 95% or higher. For lower resolution (i.e., 2.5 to 3Å) the average overall quality factor is around 91%. In this study, ERRAT calculated the overall quality factor of 97.65% for PR2 which indicated high-solution structure and 93.62% for PR3, which indicated the structure of an average resolution, respectively (Fig 5A and 5B). The predicted 3D structures of proteins are considered good if a least 80% of the residues exhibited score ≥0.2 in the 3D-ID profile [39].

Furthermore, active site prediction and identification of active residues in protein binding pockets assist in designing new drugs or finding potential targets in proteins. Molecular docking analyses using MOE has been a great way to investigate ligand-protein and protein-protein interactions. Several studies have reported the molecular interactions of chitin with chitinase enzyme and other molecular interactions which are important for plant defense strategies [54–56]. Molecular docking of extracellular chitinase from *Bacillus pumilus* MCB-7 revealed that amino acid residues Ala75, Cys98, Gln99, Val113 and Met114 interacted with chitin [54]. Moreover, chitinase from *Aspergillus fumigatus* has Trp312, Ala124, Tyr125, Tyr232 and Asn233 while chitinase from *Blastomyces dermatitidis* has Ala97, Thr98, Tyr205, Asn206, and Trp288 active residues which are involved in the protein interactions [57, 58]. In current study, chitinase protein from wheat Frontana had Gln194, Asn200, Gln238, Lys241 and Ile274 residues present in the active site pocket which were found to be involved in the interactions. Similarly, β–1,3–glucanase had Glu259, Glu307, Glu316 and Lys310 residues were found to be involved in the molecular interactions with β-glucan.

## Conclusion

Conclusively, molecular docking analysis of β–1,3–glucanase and chitinase proteins has revealed crucial amino acid residues which are involved in ligand binding and important interactions which might have important role in plant defense against various fungal pathogens. Moreover, active residues in the active sites of PR2 and PR3 proteins can also be determined through mutational studies and resulting information might help understanding how these proteins are involved in plant defense mechanisms. Lastly, the resultant information might also help to improve the plant genomic structure resulting in resistant crop and high production to meet consumer demand.

## Supporting information

S1 File (PDF)
