# Supplementary material for: Phylogenetic analyses, protein modeling and active site prediction of two pathogenesis related (PR2 and PR3) genes from bread wheat
Source: PLoS One. 2021 Sep 10;16(9):e0257392. doi: 10.1371/journal.pone.0257392 (PMC8432781; doi:10.1371/journal.pone.0257392)
Supplement: S1 File — (PDF) [file pone.0257392.s001.pdf]

S1 File

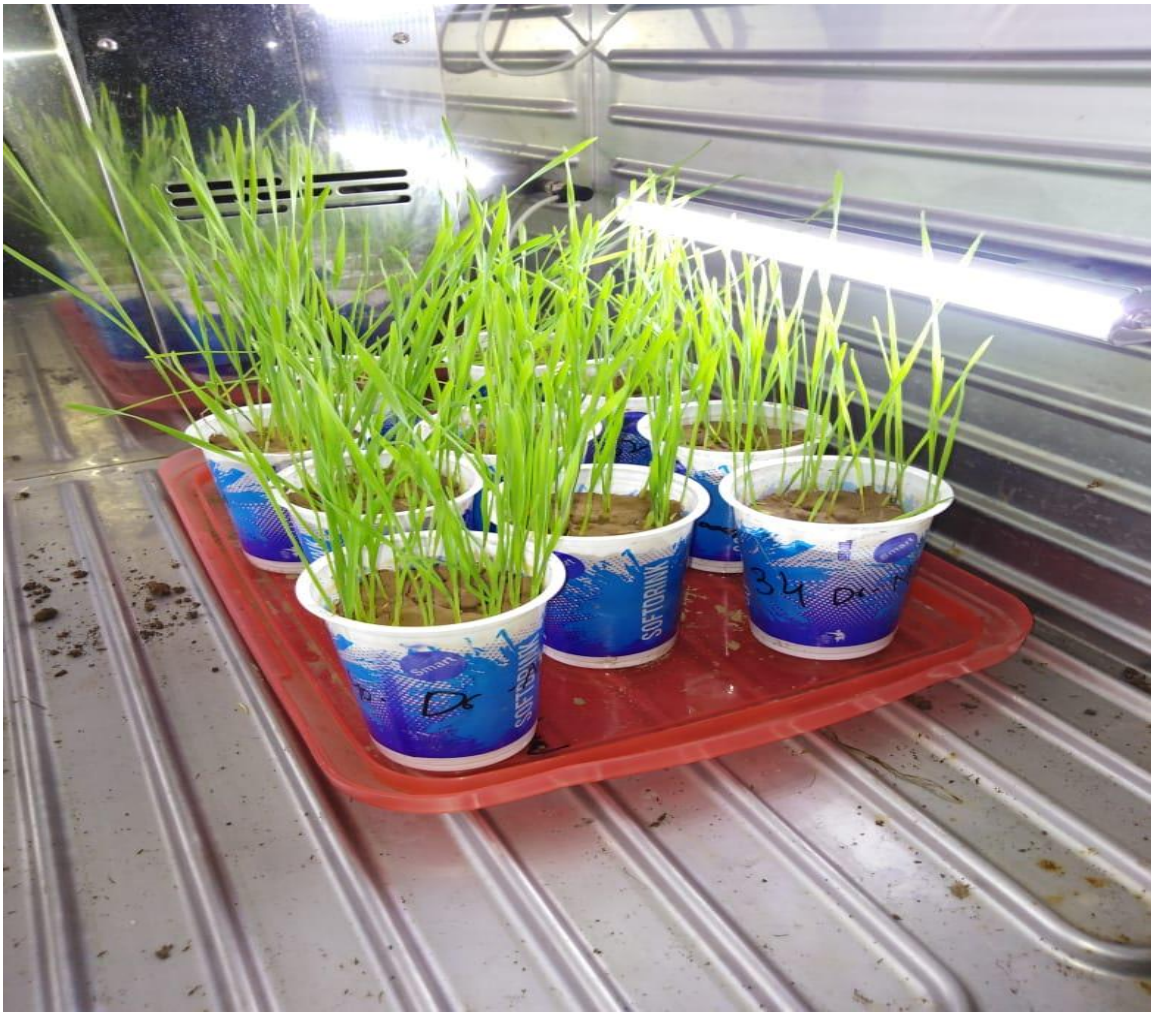

S1 Fig. Nine different wheat varieties were sown in the 4'' plastic pots. Bhakkar-2000, GA-2002, Manthar, Saleem 2000, Pirsabak-2005, Chenab-70, Chenab-97, Frontana, Punjab-76

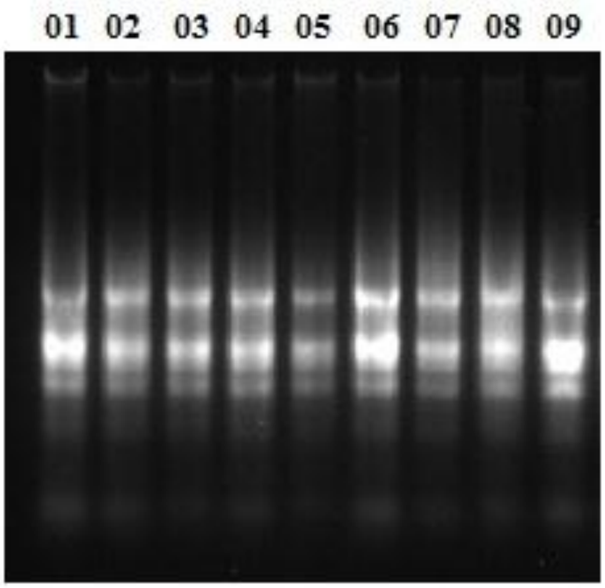

S2 Fig. RNA extraction from nine wheat varieties. Lane 01 = Chenab-70, Lane 02 = Chenab-97, Lane 03 = Bhakkar-2000, Lane 04 = GA-2002, Lane 05 =Manthar, Lane 06 = Saleem 2000, Lane 07 = Pirsabak-2005, Lane 08 = Frontana, and Lane 09 = Punjab-76

A

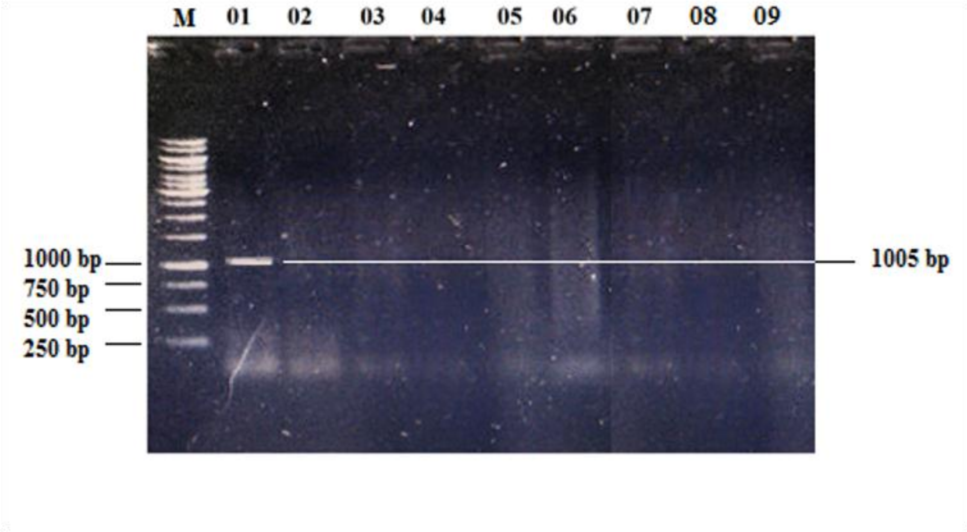

B

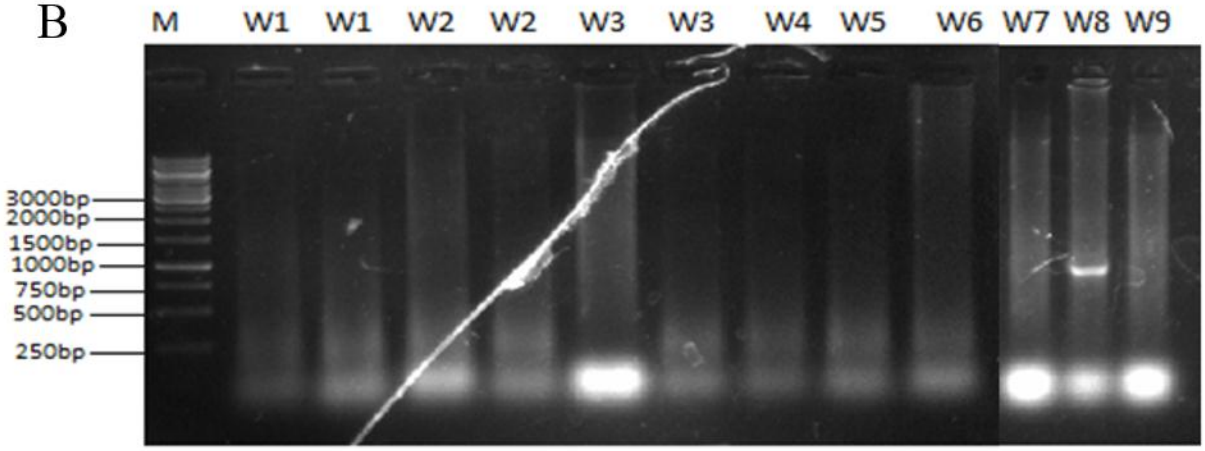

S3 Fig. Uncropped images of full-length gene amplifications of PR2 and PR3 genes. A) Lane 01 = Chenab-70, Lane 02 = Chenab-97, Lane 03 = Bhakkar-2000, Lane 04 = GA-2002, Lane 05 =Manthar, Lane 06 = Saleem 2000, Lane 07 = Pirsabak-2005, Lane 08 = Frontana, and Lane 09 = Punjab-76. **B)** W1 = Chenab-70, W2 = Chenab-97, W3 = Bhakkar-2000, W4 = GA-2002, W5 =Manthar, W6 = Saleem 2000, W7 = Pirsabak-2005, W8 = Frontana, and W9 = Punjab-76

(a)

Frame (sense)	Family	Description	Entry type	Clan	Envelope	
					Start	End
2 (+)	Glyco_hydro_17	Glycosyl hydrolases family 17	Domain	CL0058	32	337

```
#HMM     igvnyGvkgnnlPstskvislyksnnikelrlyksdtevlkalaasnlevilnlpnsdlaelesnqskaavWvqdnvrpaaekvkikyiavgneispstsaakavflvpamanirnaltaaglsnkikvstalaldildssyppsqstfreev
#MATCH   igv+yGv+gnnlPs s+v++ly+s+ i+ +r+y++d ++l al++s++  il+++n +l +++  +s aa+Wvq+nvrp++++v+ikyia+gne++ +++++    +vpam+n+++ l+aagls  ikvst++  d + +s+pps + f + +
#PP      8***********************************************************************************************999999....9*****************6.*************************99
#SEQ     IGVCYGVIGNNLPSRSDVVQLYRSKGINGMRIYFADGQALSALRNSGIGLILDIGNDQLSNIAASTSNAASWVQNNVRPYYPAVNIKYIAAGNEVQGGDTQS----IVPAMRNLNAVLSAAGLSA-IKVSTSIRFDAVANSFPPSAGVFAQSY
```

Alignment		HMM		HMM length	Bit score	E-value	Predicted active sites	Show/hide alignment
Start	End	From	To					
32	337	1	313	313	350.4	1.1e-104	n/a	Hide

```
kpvvdpivvflagtnspllvnvyPYfvysndpsnvsLdyalfqkqslvndn..glayrnlfDaildavyaaqekkGgtdielvvsEtGWPsdGstgaslenaatyveNlikkvkeGtpskpgkgietyvfalFdEnqKpeesvekhfGvfkpdkslkYsldf
   +  +  +la+t++pll+nvyPYf+y ++p ++sL+ya fq  ++v d+  gl+y +lfDa++davyaa ek+G + +++v+sE+GWPs+G ++as +na ty++ li++v  Gtp+k+   ++ety+fa+F+EnqK ++++e+ fG+f+pdks+ Y+++f
...999**************************************9999965559*******************************************************************8.*************************************98
---MTDVARLLASTGAPLLANVYPYFAYRDNPRDISLNYATFQPGTTVRDQnnGLTYTSLFDAMVDAVYAALEKAGAPGVKVVISESGWPSAGGFAASADNARTYNQGLINHVGGGTPKKRQ-ALETYIFAMFNENQKTGDATERSFGLFNPDKSPAYNIQF
```

(b)

Frame (sense)	Family	Description	Entry type	Clan	Envelope	
					Start	End
1 (+)	Glyco_hydro_19	Chitinase class I	Domain	CL0037	81	312

```
#HMM     ivsrslfeqllkhrnddacpakgfytydafiaaaksfpgfgttGdddtrkkeiaaflaqtshettGgsataedgpyawGycfvkekgassdycessakwplaagkkyyGrGpiqlsy
#MATCH   i+s+slf+q+l hrnd+ac akgfy y af+aaa+sf+gf+ttG +d rk+e+aaflaqtshettGg+ ta+dgpy+wGycf++e+ga+sdyc+++++wp+a+gkky+GrGpiq+s+
#PP      79*******************************************************************************************************************
#SEQ     IISQSLFDQMLLHRNDAACLAKGFYNYGAFVAAANSFSGFATTGSTDVRKREVAAFLAQTSHETTGGWPTAPDGPYSWGYCFNQERGATSDYCSPNSQWPCAPGKKYFGRGPIQISH
```

Alignment		HMM		HMM length	Bit score	E-value	Predicted active sites	Show/hide alignment
Start	End	From	To					
81	312	1	232	232	433.1	3.0e-130	n/a	Hide

```
nynygeaGqaiGqdllnnpdlvatdavvsfktalwfwmtaqspkpschdviiGqwspseadaaagrvpGyGvitniingglecgkgedtrvedrigfyrrycdilgvdpgdnldc
nynyg+aGqaiG+dllnnpdlva+da+vsfktalwfwmt+qspkps+hdvi+G+wsps ad+aagrvpGyGvitniingglecg+g+d rv+drigfy+rycd+lgv++gdnldc
*******************************************************************************************************************
NYNYGPAGQAIGTDLLNNPDLVASDATVSFKTALWFWMTPQSPKPSSHDVITGRWSPSGADQAAGRVPGYGVITNIINGGLECGRGQDGRVADRIGFYKRYCDLLGVSYGDNLDC
```

S4 Fig. Domain analysis of both PR2 and PR3 proteins predicted by Pfam server. (a) PR2 protein was found to be the member of Glycosyl hydrolases 17 family; **(b)** PR3 protein was found to be the member of Chitinase class I family.

S1 Table. Primer sets used to amplify PR2 and PR3 genes

Sr. No.	Primer name	Primer sequence (5′ – 3′)	Size (bp)
1	PR2-F	ATGGCTGGAAAGGATGTTG	19
2	PR2-R	TTAGAACTGGATGTTGTAG	19
3	PR3-F	ATGAGAGGAGTTGTGGTGGTG	21
4	PR3-R	CTATGCGAACGGCCTCTGGT	20
